# Coverage and Estimated Effectiveness of mRNA COVID-19 Vaccines Among US Veterans

**DOI:** 10.1001/jamanetworkopen.2021.28391

**Published:** 2021-10-06

**Authors:** Yinong Young-Xu, Caroline Korves, Jeff Roberts, Ethan I. Powell, Gabrielle M. Zwain, Jeremy Smith, Hector S. Izurieta

**Affiliations:** 1 White River Junction Veterans Affairs Medical Center, White River Junction, Vermont; 2 Geisel School of Medicine at Dartmouth, Hanover, New Hampshire; 3 Office of Vaccines Research and Review, Center for Biologics Evaluation and Research, Food and Drug Administration, White Oak, Maryland

## Abstract

**Importance:**

Effectiveness of mRNA vaccinations in a diverse older population with high comorbidity is unknown.

**Objectives:**

To describe the scope of the COVID-19 vaccination rollout among US veterans, and to estimate mRNA COVID-19 vaccine effectiveness (VE) as measured by rates of SARS-CoV-2 infection.

**Design, Setting, and Participants:**

This matched test–negative case-control study was conducted using SARS-CoV-2 test results at Veterans Health Administration sites from December 14, 2020, to March 14, 2021. Vaccine coverage was estimated for all veterans. VE against SARS-CoV-2 infection and COVID-19–related hospitalization and death were estimated using electronic health records from veterans who routinely sought care at a VHA facility and had a test result positive for SARS-CoV-2 (cases) or negative for SARS-CoV-2 (controls). Cases and controls were matched on time of test and geographic region. Data were analyzed from May to July 2021.

**Exposures:**

Vaccination status, defined as unvaccinated, partially vaccinated (≥14 days after first dose until second dose), or fully vaccinated (≥14 days after second dose), at time of test.

**Main Outcomes and Measures:**

The main outcome of interest was a positive result for SARS-CoV-2 on a polymerase chain reaction or antigen test. Secondary outcomes included COVID-19–related hospitalization and death, defined by discharge data and proximity of event to positive test result. VE was estimated from odds ratios for SARS-CoV-2 infection with 95% CIs.

**Results:**

Among 6 647 733 veterans included (3 350 373 veterans [50%] aged ≥65 years; 6 014 798 [90%] men and 632 935 [10%] women; 461 645 Hispanic veterans of any race [7%], 1 102 471 non-Hispanic Black veterans [17%], and 4 361 621 non-Hispanic White veterans [66%]), 1 363 180 (21%) received at least 1 COVID-19 vaccination by March 7, 2021. In this period, during which the share of SARS-CoV-2 variants Alpha, Epsilon, and Iota had started to increase in the US, estimates of COVID-19 VE against infection, regardless of symptoms, was 95% (95% CI, 93%-96%) for full vaccination and 64% (95% CI, 59%-68%) for partial vaccination. Estimated VE against COVID-19–related hospitalization for full vaccination was 91% (95% CI 83%-95%); there were no deaths among veterans who were fully vaccinated. VE against infection was similar across subpopulations (non-Hispanic Black, 94% [95% CI, 88%-97%]; Hispanic [any race], 83% [95% CI, 45%-95%]; non-Hispanic White, 92% [95% CI 88%-94%]; rural, 94% [95% CI, 89%-96%]; urban, 93% 95% CI, 89%-95%]).

**Conclusions and Relevance:**

For veterans of all racial and ethnic subgroups living in urban or rural areas, mRNA vaccination was associated with substantially decreased risk of COVID-19 infection and hospitalization, with no deaths among fully vaccinated veterans.

## Key Points

**Question:**

What was the COVID-19 vaccination coverage and estimated mRNA COVID-19 vaccine effectiveness (VE) among US veterans in the first 3 months following vaccine rollout?

**Findings:**

In this case-control study including 6 647 733 veterans, 23% of veterans received at least 1 COVID-19 vaccination during the first 3 months of vaccine rollout. VE against infection was estimated to be 95% for full vaccination; estimated VE against COVID-19-related hospitalization was 91%, and there were no COVID-19–related deaths among fully vaccinated veterans.

**Meaning:**

These findings suggest that early vaccination rollout for veterans was efficient, and estimated VE was high for this diverse US population.

## Teaser

This case-control study describes the scope of the mRNA COVID-19 vaccination rollout and estimates the association between vaccination and reductions in SARS-CoV-2 infections among veterans during the first 3 months of vaccine rollout.

## Introduction

On December 11, 2020, the US Food and Drug Administration (FDA) issued an Emergency Use Authorization (EUA) for the BNT162b2 COVID-19 vaccine (Pfizer-BioNTech), for the prevention of COVID-19 for individuals ages 16 years and older.^1^ One week later, the FDA issued an EUA for the mRNA-1273 COVID-19 vaccine (Moderna) for individuals aged 18 years and older^2^; both vaccines are mRNA vaccines. Prior to these EUAs, the pandemic’s impact on veterans enrolled in the Veterans Health Administration (VHA), like elsewhere, was devastating. Approximately 207 000 COVID-19 cases had been reported by the end of 2020 among VHA-enrolled veterans.^3^ Additionally, the burden of COVID-19 cases was distributed disproportionally among racial and ethnic minority populations, such as Black and Hispanic individuals, and could not be explained by underlying chronic conditions. Although the VHA patient population includes approximately 13% Black veterans and 7% Hispanic veterans, among veterans with COVID-19, approximately 35% were Black, and 13% were Hispanic. More than 10 000 of VHA patients died.^3^

The VHA worked closely with the Centers for Disease Control and Prevention and other federal partners to provide COVID-19 vaccines to veterans quickly and safely. On March 24, 2021, the success of the VHA vaccination program prompted the US Congress to pass the “Strengthening and Amplifying Vaccination Efforts to Locally Immunize All Veterans and Every Spouse Act” which authorized the Department of Veterans Affairs (VA) to expand its vaccination effort beyond VHA-enrolled veterans.^4^

Rapid deployment of the vaccination program was accompanied by VHA’s nationwide SARS-CoV-2 testing effort, which aimed to test and record both symptomatic and asymptomatic veterans across all VHA facilities. Combined with accurate and timely recording of vaccination, this created an opportunity for a robust and well-powered test-negative design (TND) case-control analysis.

Aiming to describe the extent of vaccination coverage and estimate associated reductions in SARS-CoV-2 infection that may serve as a marker of effectiveness of both mRNA vaccines in a diverse population that included individuals who were socioeconomically disadvantaged and medically high-risk, this study focused on the first 3 months of the vaccination effort in the VHA, thus the analysis excluded data on the JNJ-78436735 COVID-19 vaccine (Johnson & Johnson).

## Methods

This case-control study was approved by the institutional review board of the VA Medical Center in White River Junction, Vermont, and was granted an exemption for consent because it was deemed impractical to require consent for this minimal risk exposure. This study followed the Strengthening the Reporting of Observational Studies in Epidemiology (STROBE) reporting guideline.

### Data Source

The VHA is the largest integrated health care system in the US, providing comprehensive care to more than 9 million veterans at more than 171 medical centers and 1112 outpatient sites of care.^4^ Electronic medical record data from the VHA Corporate Data Warehouse (CDW) were analyzed. The Corporate Data Warehouse contains patient-level information on all patient encounters, treatments, prescriptions (including vaccinations), and laboratory results rendered in VHA medical facilities.

### Study Design

First, vaccination coverage, defined as having at least 1 COVID-19 vaccination administered at a VHA facility between December 14, 2020, and March 7, 2021, was described for the population of VHA enrollees. Second, a TND case-control study was conducted to estimate mRNA COVID-19 VE against infection, irrespective of symptoms and to evaluate VE against COVID-19–related hospitalization and death. The study population included veterans ages 18 years and older, with residence in a US state or Washington, District of Columbia, who presented for SARS-CoV-2 polymerase chain reaction (PCR) or antigen testing at a VHA outpatient or emergency department facility, or had testing within 1 day of hospitalization, during the study period (December 14, 2020, through March 7, 2021); veterans may have presented for testing for any reason, such as screening prior to medical procedure or for employment or travel, and they could have been symptomatic or asymptomatic. Veterans were required to have had VHA enrollment for at least 2 years prior to the study period and at least 1 inpatient or 2 outpatient visits in the past 2 years. Individuals meeting any of the following criteria were excluded: a COVID-19 diagnosis and/or positive results for SARS-CoV-2 on an antigen or PCR test at any time between February 2020 and study initiation (December 14, 2020); hospitalization more than 1 day prior to testing; or incomplete VHA medical records.

For the TND case-control study to estimate VE against infection, positive SARS-CoV-2 tests from qualifying veterans were classified as cases. Negative SARS-CoV-2 tests from qualifying veterans served as controls, and a maximum of 4 controls were matched to each case (in the main and stratum-specific analyses) based on Health and Human Services geographic region, and testing date (within 21 days of case testing date) since both factors are related to local disease burden, likelihood of having a positive SARS-CoV-2 test result, and vaccine exposure status. Because the test was the unit of analysis, more than 1 SARS-CoV-2 test per veteran was permitted for inclusion. A TND case-control study to estimate VE against symptomatic disease was also conducted. Within the population of veterans with a SARS-CoV-2 test performed, a case-control study was also conducted to estimate VE against COVID-19–related hospitalization and death. For these analyses, cases were veterans who had positive test results and were subsequently hospitalized (or died within 30 days following testing positive), and controls were veterans who were tested and did not have the outcome of interest. Up to 4 controls were matched to each case based on geographic region and testing date.

### Exposure, Outcome, and Covariate Assessment

Vaccination status was based on records of mRNA COVID-19 vaccination at a VHA facility from December 14, 2020, to February 28, 2021. A veteran was classified as unvaccinated until the day prior to the first vaccination, partially vaccinated from day 14 after the first vaccination until the day prior to the second vaccination, and fully vaccinated starting at 14 days after the second vaccination (eFigure in the Supplement). Days 0 to 13 after the first and second doses were excluded from all analyses. Vaccination status was determined at the date of testing.

For the TND case-control study to estimate VE against infection, medical records were used to identify cases (positive tests) and controls (negative tests). For the TND case-control study to assess VE against symptomatic disease, veterans who had positive test results were further restricted to those who, on the day of testing, had evidence of at least 1 COVID-19 symptom (eTable 1 in the Supplement). For the case-control study for hospitalization and death, a COVID-19–related hospitalization was identified by the presence of an admission and discharge diagnosis of COVID-19 occurring any time after the first positive SARS-CoV-2 test result. A death occurring in hospital with a COVID-19 discharge diagnosis or a death occurring within 30 days of a positive SARS-CoV-2 test result was classified as a COVID-19–related death; controls were drawn from veterans with a positive result on a SARS-CoV-2 test who were not hospitalized for COVID-19 during the study period (or who did not die within 30 days of SARS-CoV-2 test).

Demographic and clinical characteristics of patients tested for SARS-CoV-2 were assessed at the time of testing or based on data from the prior 2-year period, for characteristics such as comorbidities, Charlson Comorbidity Index and Care Assessment Needs score. A variable for race and ethnicity was determined from separate variables for race and ethnicity captured in the medical record so that outcomes could be assessed for these subgroups (eTable 1 in the Supplement).

### Statistical Analysis

For VHA enrollees and for subpopulations, vaccination coverage was reported as the frequency and proportion of individuals with at least 1 vaccination. For the TND case-control study, for cases (positive SARS-CoV-2 test result) and controls (negative SARS-CoV-2 test result), baseline demographic and clinical characteristics of the tested veterans were described by reporting frequency and proportion for categorical variables and mean (SD) for continuous variables. Missing data were reported or included within a level of a categorical variable as indicated in eTable 1 in the Supplement (eg, patient with no record of a specific comorbidity was assumed to not have the comorbidity). Standardized mean differences^5^ were used to describe differences in characteristics between cases and controls. Conditional logistic regression was used to calculate odds ratios (ORs) with 95% CIs for the association between positive SARS-CoV-2 testing and receipt of mRNA COVID-19 vaccine. VE was estimated as (1 − *OR_odds of SARS-CoV-2 in vaccinated vs odds of SARS-CoV-2 in unvaccinated_*) × 100%. Analyses were conducted comparing full vaccination and partial vaccination vs no vaccination; sensitivity analyses of time since vaccination were also conducted. Models for adjusted analyses included covariates for potential confounders of the association between vaccination status and having positive test results for SARS-CoV-2. Confounders were determined a priori based on known factors associated with SARS-CoV-2 testing and test positivity in the VA population^6^ and factors associated with COVID-19 risk and vaccination.^7,8^ Analyses were conducted for patient subgroups, including stratifications by age, race and ethnicity, and rural vs urban location. Conditional logistic regression for the case-control analyses of hospitalization and death and for VE against symptomatic disease were conducted similarly.

All analyses were conducted using SAS statistical software version 9.4 (SAS Institute). *P* values were 2-sided, and statistical significance was set at *P* < .05. Data were analyzed from May to July 2021.

## Results

### Vaccination Coverage

Of 6 647 733 VHA enrollees included (3 350 373 veterans [50%] aged ≥65 years; 6 014 798 [90%] men and 632 935 [10%] women; 461 645 Hispanic veterans of any race [7%], 1 102 471 non-Hispanic Black veterans [17%], and 4 361 621 non-Hispanic White veterans [66%]), 1 363 180 veterans (20%) had been administered at least 1 dose of a COVID-19 vaccine at a VHA facility by March 7, 2021 (Table 1). Vaccination coverage was higher (1 082 417 veterans [32%]) among veterans ages 65 years and older. Vaccination coverage for this early vaccination period was 461 645 Hispanic veterans of any race (18%), 234 363 Black veterans (21%), and 940 691 White veterans (22%). Vaccination reached 24 103 of 124 850 veterans experiencing homelessness (19%) and 11 675 of 29 176 veterans living in nursing homes (40%) (Table 1).

**Table 1.** COVID-19 Vaccination Coverage (≥1 Dose) Through March 7, 2021, Among VHA Enrollees

Characteristic	Enrollees, No.	Vaccinated enrollees, No. (%)
All enrollees	6 647 733	1 363 180 (21)
Age range, y		
18-44	1 368 610	38 504 (3)
45-64	1 928 750	242 259 (13)
65-74	1 867 540	570 131 (31)
75-84	1 009 567	363 245 (36)
≥85	473 266	149 041 (32)
≥65	3 350 373	1 082 417 (32)
Sex		
Women	632 935	74 795 (12)
Men	6 014 798	1 288 385 (21)
Race and ethnic group		
Hispanic (any race)	461 645	81 480 (18)
Non-Hispanic Black	1 102 471	234 363 (21)
Non-Hispanic White	4 361 621	940 691 (22)
Other^a^	381 648	70 080 (18)
Missing	340 348	36 566 (11)
Urban-rural classification^b^		
Highly rural	82 514	11 985 (15)
Rural	2 154 833	387 112 (18)
Urban	4 410 386	964 083 (22)
VHA-defined priority group^b^		
1-4	2 527 976	478 364 (19)
5-6	2 136 265	417 575 (20)
7-8	1 983 492	467 241 (24)
Nursing home resident	29 176	11 675 (40)
Homeless	124 850	24 103 (19)
Low income	663 649	138 463 (21)
Comorbidities^b^		
Asthma	329 307	122 736 (37)
Cancer (any)	186 911	85 194 (46)
Cancer (metastatic)	18 963	9789 (52)
Coronary artery disease	359 302	150 120 (42)
Congestive heart failure	160 639	69 824 (44)
Chronic kidney disease	205 473	90 655 (44)
Chronic obstructive pulmonary disease	355 011	132 668 (37)
Cardiovascular disease	111 379	44 097 (40)
Dementia	67 493	24 143 (36)
Dyslipidemia	735 152	260 834 (36)
HIV	19 279	6703 (35)
Hypertension	1 453 671	517 021 (36)
Liver disease		
Mild	92 629	32 212 (35)
Severe	7651	2951 (39)
Myocardial infarction	35 230	15 134 (43)
Hemiplegia or paraplegia	19 531	8342 (43)
Peptic ulcer disease	8425	3396 (40)
Peripheral vascular disease	136 248	60 224 (44)
Rheumatoid arthritis	42 925	15 939 (37)
Renal disease	212 003	93 517 (44)
Immunocompromised	445 973	190 215 (43)
Diabetes	953 054	347 594 (37)

Abbreviation: VHA, Veterans Health Administration.

^a^ Other race or ethnicity included Asian and American Indian or Alaska Native veterans.

^b^ Definitions for urban-rural classification, VHA defined priority group, and comorbidities are provided in eTable 1 in the Supplement.

### Study Population for VE

There were 15 110 veterans with positive SARS-CoV-2 test results, classified as cases, and 472 452 veterans with negative test results, classified as controls (Figure). After matching, there were 15 110 cases and 60 436 controls. Baseline characteristics for cases and controls before and after matching are shown in Table 2. At the time of testing, 14 799 cases (98%) and 53 075 controls (88%) were unvaccinated in the matched analysis. Compared with controls, cases were younger and more likely to be White.

**Figure.**
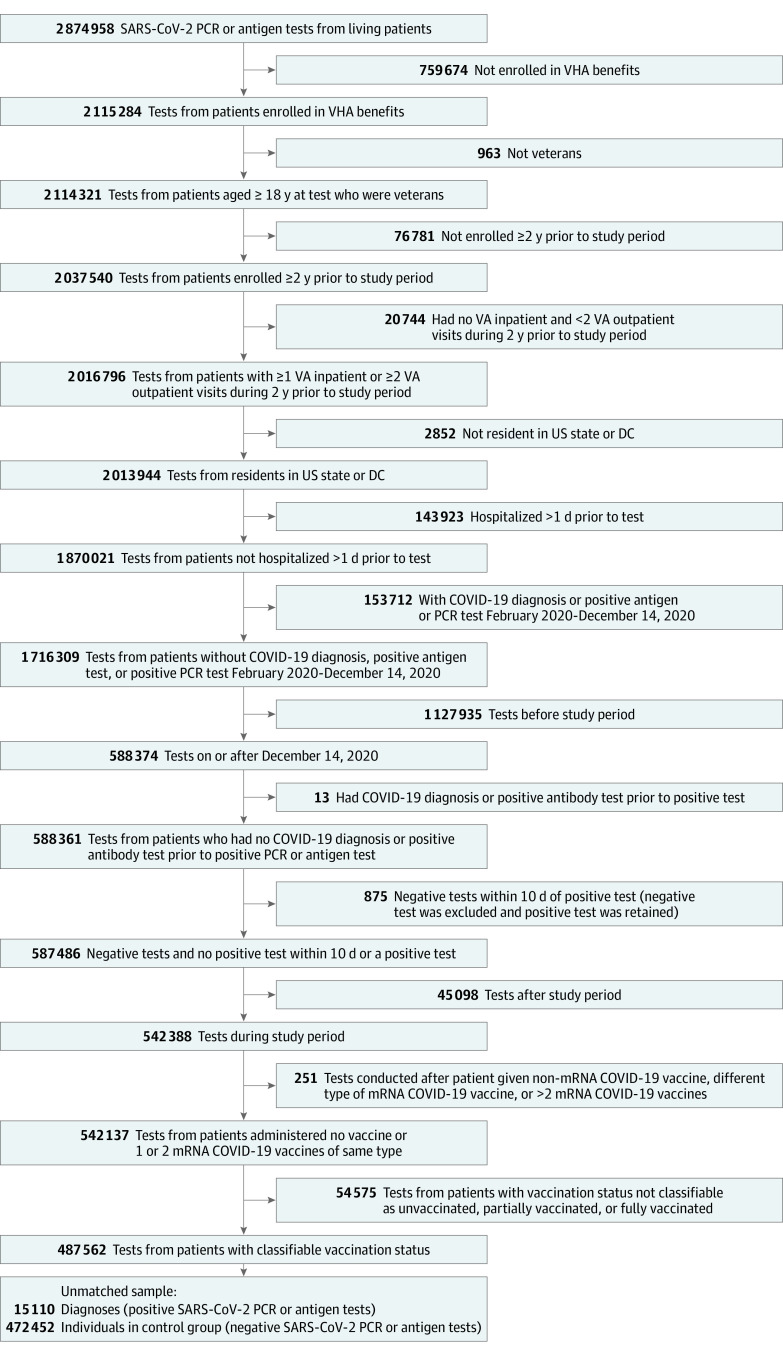
Veterans With SARS-CoV-2 Polymerase Chain Reaction (PCR) and Antigen Tests (December 14, 2020-March 7, 2021) Meeting Study Criteria DC indicates District of Columbia; VA, Department of Veterans Affairs; and VHA, Veterans Health Administration.

**Table 2.** Exposure Status and Baseline Characteristics of Study Participants^a^

Characteristic	Unmatched			Matched		
	No. (%)		Standardized difference^b^	No. (%)		Standardized difference^b^
	Cases (n = 15 110)	Controls (n = 472 452)		Cases (n = 15 110)	Controls (n = 60 436)	
Vaccination status						
Full	41 (0)	24 923 (5)	31	41 (0)	3711 (6)	34
Partial	270 (2)	21 469 (5)	16	270 (2)	3650 (6)	22
None	14 799 (98)	426 060 (90)	–33	14 799 (98)	53 075 (88)	–40
Vaccine manufacturer						
Moderna	192 (1)	26 142 (6)	24	192 (1)	4362 (7)	30
Pfizer-BioNTech	119 (1)	20 250 (4)	22	119 (1)	2999 (5)	25
None (unvaccinated)	14 799 (98)	426 060 (90)	–33	14 799 (98)	53 075 (88)	–40
Month of test						
December 2020	4594 (30)	109 631 (23)	–16	4594 (30)	18 403 (30)	0
January 2021	7190 (48)	182 025 (39)	–18	7190 (48)	24 939 (41)	–13
February 2021	2826 (19)	143 442 (30)	27	2826 (19)	14 015 (23)	11
March 2021	500 (3)	37 354 (8)	20	500 (3)	3079 (5)	9
Care setting for test						
ED or at admission	6812 (45)	114 297 (24)	–45	6812 (45)	16 176 (27)	–39
Outpatient	8298 (55)	358 155 (76)	45	8298 (55)	44 260 (73)	39
Age group, y						
18-44	2533 (17)	72 765 (15)	–4	2533 (17)	711 (1)	–57
45-64	5358 (35)	173 499 (37)	3	5358 (35)	23 011 (38)	5
65-74	4634 (31)	148 917 (32)	2	4634 (31)	23 919 (40)	19
75-84	1840 (12)	57 506 (12)	0	1840 (12)	9003 (15)	8
≥85	745 (5)	19 765 (4)	–4	745 (5)	3792 (6)	6
Sex						
Women	1447 (10)	54 404 (12)	6	1447 (10)	5948 (10)	1
Men	13 663 (90)	418 048 (88)	–6	13 663 (90)	54 488 (90)	–1
Race and ethnicity						
Hispanic any race	1000 (7)	33 556 (7)	2	1000 (7)	2818 (5)	–8
Non-Hispanic Black	3776 (25)	116 024 (25)	–1	3776 (25)	19 525 (32)	16
Non-Hispanic White	9430 (62)	286 753 (61)	–4	9430 (62)	34 036 (56)	–12
Other^c^	904 (6)	36 119 (8)	7	904 (6)	4057 (7)	3
Rurality						
Highly rural	106 (1)	2886 (1)	–1	106 (1)	381 (1)	–1
Rural	4590 (30)	114 688 (24)	–14	4590 (30)	14 506 (24)	–14
Urban	10 414 (69)	354 878 (75)	14	10 414 (69)	45 549 (75)	14
HHS region						
1	788 (5)	21 819 (5)	–3	788 (5)	3152 (5)	0
2	737 (5)	22 027 (5)	–1	737 (5)	2948 (5)	0
3	2242 (15)	46 158 (10)	–15	2242 (15)	8967 (15)	0
4	3921 (26)	131 404 (28)	4	3921 (26)	15 684 (26)	0
5	1629 (11)	63 531 (13)	8	1629 (11)	6516 (11)	0
6	3356 (22)	55 827 (12)	–28	3356 (22)	13 421 (22)	0
7	705 (5)	22 340 (5)	0	705 (5)	2820 (5)	0
8	434 (3)	15 182 (3)	2	434 (3)	1736 (3)	0
9	966 (6)	76 926 (16)	32	966 (6)	3864 (6)	0
10	332 (2)	17 212 (4)	9	332 (2)	1328 (2)	0
Homeless	609 (4)	31 348 (7)	12	609 (4)	4595 (8)	15
Low income	2100 (14)	74 032 (16)	5	2100 (14)	11 912 (20)	16
Nursing home use	118 (1)	7966 (2)	8	118 (1)	1792 (3)	16
VA priority group						
1-4	5713 (38)	198 875 (42)	9	5713 (38)	31 344 (52)	29
5-6	5043 (33)	153 490 (32)	–2	5043 (33)	17 016 (28)	–11
7-8	4354 (29)	120 087 (25)	–8	4354 (29)	12 076 (20)	–21
Quan CCI, mean (SD)	1.3 (1.9)	1.5 (2.1)	10	1.3 (1.9)	2.0 (2.4)	34
Overweight or obese	7515 (50)	216 362 (46)	–8	7515 (50)	26 825 (44)	–11
Comorbidities						
Asthma	1677 (11)	63 472 (13)	7	1677 (11)	11 427 (19)	22
Cancer (any)	950 (6)	37 468 (8)	6	950 (6)	5720 (9)	12
Cancer (metastatic)	140 (1)	7068 (1)	5	140 (1)	1082 (2)	7
Coronary artery disease	1954 (13)	62 707 (13)	1	1954 (13)	10 502 (17)	12
Congestive heart failure	1153 (8)	41 766 (9)	4	1153 (8)	7488 (12)	16
Chronic kidney disease	1396 (9)	46 036 (10)	2	1396 (9)	8180 (14)	14
Chronic obstructive pulmonary disease	1811 (12)	69 016 (15)	8	1811 (12)	12 365 (20)	23
Cardiovascular disease	653 (4)	27 221 (6)	7	653 (4)	5055 (8)	17
Dementia	473 (3)	26 253 (6)	12	473 (3)	5807 (10)	27
Diabetes (complicated)	1766 (12)	53 579 (11)	–1	1766 (12)	9467 (16)	12
Diabetes (uncomplicated)	2773 (18)	73 168 (15)	–8	2773 (18)	11 423 (19)	1
Dyslipidemia	3467 (23)	113 138 (24)	2	3467 (23)	18 058 (30)	16
HIV	80 (1)	3857 (1)	4	80 (1)	862 (1)	9
Hypertension	6524 (43)	202 022 (43)	–1	6524 (43)	32 880 (54)	23
Liver disease						
Mild	563 (4)	22 694 (5)	5	563 (4)	3945 (7)	13
Moderate or severe	78 (1)	3533 (1)	3	78 (1)	554 (1)	5
Myocardial infarction (history)	329 (2)	12 093 (3)	3	329 (2)	2015 (3)	7
Peripheral vascular disease	861 (6)	33 606 (7)	6	861 (6)	6054 (10)	16
Rheumatoid arthritis	212 (1)	6698 (1)	0	212 (1)	1076 (2)	3
Renal disease	1429 (9)	46 809 (10)	2	1429 (9)	8303 (14)	13
Atrial fibrillation	1158 (8)	39 164 (8)	2	1158 (8)	6303 (10)	10
Arthritis	1336 (9)	44 914 (10)	2	1336 (9)	7987 (13)	14
Depression	2224 (15)	81 741 (17)	7	2224 (15)	11 703 (19)	12
Embolism (history)	295 (2)	11 705 (2)	4	295 (2)	2051 (3)	9
Hyperlipidemia	4502 (30)	140 785 (30)	0	4502 (30)	22 315 (37)	15
Musculoskeletal disorder	7225 (48)	240 191 (51)	6	7225 (48)	35 050 (58)	20
Obesity	1685 (11)	51 077 (11)	–1	1685 (11)	7209 (12)	2
Parkinson	115 (1)	6524 (1)	6	115 (1)	1117 (2)	10
Sickle cell disease	11 (<1)	358 (<1)	0	11 (<1)	77 (<1)	2
Solid organ transplant recipient	68 (<1)	2492 (1)	1	68 (<1)	389 (1)	3
Stroke or transient ischemic attack	329 (2)	14 799 (3)	6	329 (2)	2731 (5)	13
Urinary tract infection	360 (2)	16 923 (4)	7	360 (2)	3300 (5)	16
Immunocompromised	2236 (15)	89 287 (19)	11	2236 (15)	14 079 (23)	22
CAN score, mean (SD), %						
Mortality						
1 y	5.1 (10.1)	5.4 (10.4)	3	5.1 (10.1)	7.4 (11.9)	20
90 d	1.5 (3.9)	1.6 (4.0)	3	1.5 (3.9)	2.2 (4.7)	16
Event						
1 y	20.4 (20.2)	20.6 (20.9)	1	20.4 (20.2)	24.6 (23.4)	19
90 d	7.9 (11.4)	8.3 (11.8)	3	7.9 (11.4)	10.3 (13.8)	19
Hospitalization						
1 y	17.7 (17.7)	18.6 (18.9)	5	17.7 (17.7)	22.1 (21.2)	22
90 d	7.2 (10.0)	7.8 (10.9)	6	7.2 (10.0)	9.7 (12.7)	22
Any COVID-19 symptoms	10 296 (68)	101 167 (21)	–106	10 296 (68)	12 901 (21)	–107
Influenza vaccination						
2019-2020 season	131 (1)	4252 (1)	0	131 (1)	719 (1)	3
2020-2021 season	1517 (10)	49 458 (10)	1	1517 (10)	8067 (13)	10

Abbreviations: CAN, Care Assessment Needs; CCI, Charlson Comorbidity Index; ED, emergency department; HHS, Health and Human Services; VA, Veterans Affairs.

^a^ Definitions for variables are provided in eTable 1 in the Supplement.

^b^ Standardized difference of 10 or greater was used to identify imbalance between cases and controls.

^c^ Other race or ethnicity included Asian and American Indian or Alaska Native veterans.

### Vaccine Effectiveness

Table 3 shows the estimated VE for full and partial vaccination against a documented positive SARS-CoV-2 test result, regardless of symptoms. Among the overall population, the adjusted VE for full vaccination was 95% (95% CI, 93%-96%) and the adjusted VE for partial vaccination was 64% (95% CI, 59%-68%). Estimated VE was similar to that in the overall population for most subpopulations. The point estimate of VE for full vaccination was 8 percentage points lower among veterans who were immunocompromised (87% [95% CI, 79%-92%]), and in a post hoc analysis, it was 69% (95% CI, 17%-88%) for veterans with hematological malignant neoplasms (eTable 2 in the Supplement).

**Table 3.** Estimated VE Against Laboratory-Confirmed SARS-CoV-2 Infection^a^

Characteristic	VE, % (95%CI)					Adjusted VE, % (95%CI)^b^									
	Full vs no vaccination		Partial vs no vaccination		Full vaccination					Partial vaccination				
	Unadjusted	Adjusted^b^	Unadjusted	Adjusted^b^	From 7 d after dose 2			From 14 d after dose 2		7 d after dose 1 until dose 2	14-20 d after dose 1		14 d after dose 1 until dose 2	
Overall	97 (95 to 97)	95 (93 to 96)	75 (71 to 78)	64 (59 to 68)	94 (92 to 95)			95 (93 to 96)		58 (54 to 62)	63 (57 to 69)		64 (59 to 68)	
Age group, y														
18-64	91 (82 to 96)	89 (79 to 95)	65 (52 to 75)	64 (50 to 74)	NA			NA		NA	NA		NA	
≥65	94 (91 to 96)	93 (90 to 95)	59 (52 to 64)	58 (52 to 64)	NA			NA		NA	NA		NA	
18 to 79	92 (89 to 94)	92 (89 to 94)	62 (56 to 67)	58 (52 to 64)	NA			NA		NA	NA		NA	
≥80	97 (92 to 99)	96 (91 to 98)	63 (52 to 71)	57 (44 to 67)	NA			NA		NA	NA		NA	
VHA to defined priority group														
1-4	95 (91 to 97)	94 (91 to 97)	62 (53 to 69)	61 (53 to 69)	NA			NA		NA	NA		NA	
5-6	89 (82 to 93)	89 (83 to 93)	52 (40 to 61)	55 (43 to 64)	NA			NA		NA	NA		NA	
7-8	94 (87 to 97)	94 (87 to 97)	57 (46 to 67)	59 (47 to 68)	NA			NA		NA	NA		NA	
Race and ethnic group														
Hispanic any race	83 (45 to 95)	83 (45 to 95)	46 (0 to 71)	36 (–19 to 66)	NA			NA		NA	NA		NA	
Non-Hispanic Black	94 (88 to 97)	94 (88 to 97)	55 (42 to 66)	52 (37 to 63)	NA			NA		NA	NA		NA	
Non-Hispanic White	92 (89 to 94)	92 (88 to 94)	61 (54 to 66)	58 (51 to 64)	NA			NA		NA	NA		NA	
Other^c^	NA	NA	81 (58 to 92)	80 (54 to 91)	NA			NA		NA	NA		NA	
Sex														
Women	90 (59 to 98)	86 (41 to 97)	86 (57 to 96)	87 (58 to 96)	NA			NA		NA	NA		NA	
Men	93 (90 to 95)	93 (90 to 95)	59 (53 to 64)	57 (51 to 62)	NA			NA		NA	NA		NA	
Urban/rural																
Rural	93 (88 to 96)	94 (89 to 96)	63 (54 to 71)	62 (52 to 70)	NA			NA		NA	NA		NA	
Urban	93 (90 to 95)	93 (89 to 95)	57 (51 to 63)	55 (48 to 62)	NA			NA		NA	NA		NA	
Low income	93 (83 to 97)	94 (84 to 97)	69 (54 to 79)	65 (48 to 77)	NA			NA		NA	NA		NA	
Homeless	93 (71 to 98)	94 (75 to 98)	71 (42 to 85)	69 (39 to 85)	NA			NA		NA	NA		NA	
Overweight or obese	92 (86 to 95)	91 (85 to 95)	54 (45 to 62)	52 (42 to 60)	87 (82 to 91)			91 (85 to 95)		45 (37 to 52)	58 (46 to 67)		52 (42 to 60)	
Underlying medical conditions														
Cancer	83 (66 to 91)	86 (72 to 93)	57 (37 to 71)	55 (33 to 69)	84 (73 to 91)			86 (72 to 93)		43 (25 to 56)	56 (29 to 73)		55 (33 to 69)	
Congestive heart failure	88 (79 to 93)	90 (81 to 95)	62 (47 to 73)	67 (54 to 77)	85 (76 to 90)			90 (81 to 95)		54 (42 to 64)	70 (53 to 81)		67 (54 to 77)	
Chronic kidney disease	91 (84 to 95)	92 (86 to 96)	62 (49 to 72)	63 (51 to 73)	91 (85 to 94)			92 (86 to 96)		55 (45 to 64)	66 (49 to 78)		63 (51 to 73)	
Diabetes	93 (89 to 96)	93 (89 to 96)	63 (55 to 70)	62 (54 to 69)	92 (88 to 94)			93 (89 to 96)		56 (49 to 62)	64 (53 to 72)		62 (54 to 69)	
Hypertension	92 (88 to 94)	92 (88 to 94)	59 (51 to 65)	60 (52 to 66)	91 (88 to 93)			92 (88 to 94)		56 (51 to 61)	62 (53 to 69)		60 (52 to 66)	
Immunocompromised^d^	86 (77 to 91)	87 (79 to 92)	47 (33 to 58)	51 (38 to 62)	88 (82 to 92)			87 (79 to 92)		43 (32 to 52)	57 (41 to 68)		51 (38 to 62)	

Abbreviations: NA, not applicable; VE, vaccine effectiveness; VHA, Veterans Health Affairs.

^a^ Definitions for variables are provided in eTable 1 in the Supplement.

^b^ The adjusted variables include the following: age, body mass index, cancer, congestive heart failure, chronic kidney disease, diabetes, hypertension, immunocompromised, priority level, race and ethnicity, sex, and rurality.

^c^ Other race or ethnicity included Asian and American Indian or Alaska Native veterans.

^d^ In a post hoc analysis, estimated VE against infection was 69% (95% CI 17-88) for veterans with hematological malignant neoplasms (eTable 2 in the Supplement).

VE estimates were similar across all age groups, sex, race and ethnicity, or urban vs rural status, with overlapping 95% CIs. In sensitivity analyses, the VE point estimates for partial (and full) vaccination were higher when measured starting 14 days vs 7 days after the first (and second) dose. When the analysis was restricted to symptomatic cases, VE for the overall population was 93% (95% CI, 89%-95%), approximately the same as for all tested individuals (eTable 3 in the Supplement).

The VE estimate against hospitalization was 91% (95% CI, 83%-95%). There were no deaths among veterans who were fully vaccinated (Table 4; eTable 4 in the Supplement).

**Table 4.** Estimated VE Against COVID-19–Related Hospitalization and Death^a^

Outcome	VE, % (95% CI)			
	Full vs no vaccination		Partial vs no vaccination	
	Unadjusted	Adjusted^b^	Unadjusted	Adjusted^b^
Hospitalization	89 (80-94)	91 (83-95)	41 (24-54)	48 (32-60)
Death^c^	100	100	47 (3-71)	63 (24-81)

Abbreviation: VE, vaccine effectiveness.

^a^ Definitions for variables are provided in eTable 1 in the Supplement.

^b^ The adjusted variables include the following: age, body mass index, cancer, congestive heart failure, chronic kidney disease, diabetes, hypertension, immunocompromised, priority level, race and ethnicity, sex, and rurality.

^c^ There were no deaths among fully vaccinated veterans and 644 deaths among unvaccinated veterans (eTable 4 in the Supplement).

In sensitivity analysis to assess veterans who may have received COVID-19 vaccination or treatment outside of the VHA, the analysis was repeated with Medicare data, including any records of vaccination and hospitalization outside the VHA for these veterans. The results were similar, with a VE of 95% (95% CI, 93%-96%) (eTable 5 in the Supplement). To assess potential misclassification of cases and controls due to differing sensitivities and specificities between PCR and antigen tests, the analysis of VE against infection was repeated stratifying by type of test, and there was no effect on the estimate (eTable 6 in the Supplement).

## Discussion

This case-control study found that the VHA’s vaccination effort reached all demographic groups among veterans in the first 3 months following availability of mRNA COVID vaccines under EUA. Notably, vaccination coverage was similar among non-Hispanic Black veterans and non-Hispanic White veterans. This is in contrast to a study among insured individuals in the US that reported lower coverage for non-Hispanic Black individuals (40.7%) and Hispanic individuals (41.1%) vs non-Hispanic White individuals (54.6%)^9^ and a study among the active US military that reported that non-Hispanic Black individuals were 28% less likely to initiate vaccination than non-Hispanic White individuals.^10^

In a period during which the share of SARS-CoV-2 variants Alpha, Epsilon, and Iota had started to increase in the US,^11^ this nationwide study demonstrated high VE of mRNA vaccines against both laboratory confirmed SARS-CoV-2 infections (regardless of symptoms) and symptomatic disease. Although the sample size constrained our ability to analyze subpopulations in this short study period, VE estimates were similarly high in most subpopulations, for symptomatic infections, and against COVID-19–associated hospitalization and death.

Our VE estimates are comparable to those from the clinical trials^12,13^ and from other observational studies. Two VE studies from Israel following the rollout of the BNT162b2 COVID-19 vaccine among individuals ages 16 years and older found similar results: a national surveillance data study found a VE of 95% (95% CI, 95%-96%),^14^ and a study in Israel’s largest health care organization found a VE of 92% (95% CI, 88%-95%).^15^ Results were also similar to those from a prospective study among vaccinated health care workers in the United Kingdom (VE, 85% [95% CI, 74%-96%]),^16^ a smaller study among essential and frontline workers in the US (VE, 90% [95% CI, 68%-97%]),^17^ and a recent study among US veterans (VE, 97% [95% CI, 97%-98%]).^18^

Although power was limited, the VE estimates against COVID-19 hospitalization were comparable to those from 2 studies in Israel (VE, 97% [95% CI, 97%-98%]^14^ and 87% [95% CI, 55%-100%]^15^), and to a US study of patients ages 65 years and older (VE, 94% [95% CI, 49%-99%]).^19^ Also comparable were results for VE against death, which was 97% (95% CI, 96%-97%) in a study in Israel.^14^

This case-control study using data from a large and diverse US population, which includes a large proportion of elderly individuals, adds important context to the understanding of VE because vaccination rollout and SARS-CoV-2 variants have differed from state to state and country to country. In Israel and the United Kingdom, mRNA COVID-19 vaccine distribution included only the BNT162b2 (not mRNA-1273) COVID-19 vaccine, and the United Kingdom extended the interval between doses to vaccinate their population more rapidly with 1 dose, limiting the ability to directly compare with their results.^15,20^ Furthermore, each country has distinct demographic characteristics, so this study allowed for a more in-depth analysis of VE in subpopulations for whom the COVID-19 disease burden has been greater in the US, such as Black and Hispanic individuals.

Many important prior clinical trials and observational studies^13,14,16,17,21^ had limited sample size to adequately assess the effectiveness of COVID-19 vaccines for people with underlying medical conditions, although some conditions may predispose individuals to severe consequences from infection.^22^ The immune response to vaccination among individuals who are immunocompromised has not been fully explored. When fully vaccinated, VE in our study was 87% (95% CI, 79%-92%) for veterans who were immunocompromised, which is reassuring; however, the population of patients identified as immunocompromised in this study was diverse in their immunosuppressive conditions. Given that the response to infection may vary according to underlying conditions^22^ and that a study by Lee et al^23^ reported that COVID-19 outcomes may be more severe among patients with hematological malignant neoplasms vs solid tumors, a post hoc analysis was conducted and estimated VE against infection was 69% (95% CI, 17%-88%) for this subgroup of veterans.

COVID-19 has disproportionally affected racial and ethnic minority populations and low-income communities.^24,25,26^ These results show that the VHA was able to vaccinate minority and low-income veterans with similar efficiency. Prior to vaccination, Black and Hispanic veterans were at 30% to 40% higher risk of infection compared with their White counterparts.^25^ After the VHA’s thorough effort to provide vaccination to all veterans, regardless of race, ethnicity, or socioeconomic status, the risk of SARS-CoV-2 infection (irrespective of symptoms) following full vaccination was reduced for all, with similar VE for all racial and ethnic groups, demonstrating that equitable distribution of vaccination may be an effective means to reduce racial, ethnic, and socioeconomic disparity in COVID-19 disease burden.

### Limitations

This study has some limitations. A main concern regarding test-negative studies is misclassification. This study relied on vaccination records collected prospectively in near real-time rather than using participants’ recall. While this study demonstrated that vaccination coverage reached all demographic subgroups, the coverage rates reported here may be conservative estimates,^27^ given that vaccination of individuals by state and local health departments might not have been reported to the VHA. Moreover, by the end of 2020, the VHA had standardized its testing and case definitions, unlike at the beginning of the pandemic.^28^ A TND case-control study was implemented to limit selection bias and minimize differences in health seeking behavior between cases and controls. SARS-CoV-2–positive test results coupled with COVID-19 symptoms were used in a sensitivity analysis to reduce potential misclassification and the VE from that analysis matched that of the main analysis at 93% (95% CI, 89%-95%). Some veterans vaccinated through the VHA could have been hospitalized in a non-VHA facility, especially in rural communities.^29^ The study population was limited to veterans who routinely sought care at VHA facilities to minimize likelihood of including those who were vaccinated or sought treatment for COVID-19 elsewhere. To further address this, the analysis was repeated with Medicare data, including any records of vaccination and hospitalization outside the VHA for these veterans. The results were similar with a VE of 95% (95% CI, 93%-96%). The analysis used data from both antigen and PCR tests, which may differ in their sensitivities and specificities, leading to misclassification of cases and controls. While this misclassification would likely be nondifferential, the analysis of VE against infection was repeated, stratifying by type of test, and there was no effect on the estimate. This analysis included test results from all veterans who underwent testing, without imposing strict rules to identify vaccine eligibility at the time of the test because the prevalence of conditions that would have made veterans vaccine eligible early on was quite high (eg, 55% hypertension among controls); the analysis was also conducted for subgroups of veterans who would have been vaccine-eligible early on. Nevertheless, there could still be residual misclassification and differences in health seeking behavior and disease risk between different subpopulations and for individuals in the VHA and Medicare system that would require further investigation in future studies. This analysis was conducted during the early phase of vaccination rollout, and sequencing data were not available. Future analyses of VE for variants of SARS-CoV-2 are warranted, given that there may be differences in VE for variants.^30,31,32^

## Conclusions

In this TND case-control study using data from US veterans who were tested for SARS-CoV-2, mRNA vaccines were associated with reduced risk of SARS-CoV-2 laboratory-confirmed infection, symptomatic disease, hospitalization, and death. Over a period of only 3 months after the first COVID-19 vaccine was authorized, the VHA successfully vaccinated and tested millions of veterans of all socioeconomic groups for COVID-19. The findings of this study suggest that effectiveness of these vaccines, combined with their equitable and efficient deployment, may have resulted in attenuated COVID-19 disease burden among all VHA-enrolled veterans and specific high-risk populations.

## Notes

Supplement. **eFigure.** Vaccination Exposure Classification **eTable 1.** Definitions of Variables **eTable 2.** Estimated Vaccine Effectiveness Against Laboratory-Confirmed SARS-CoV-2 Infection for Patients With Hematological Malignant Neoplasms **eTable 3.** Estimated Vaccine Effectiveness Against Laboratory-Confirmed SARS-CoV-2 Infection Among Veterans With COVID-19 Symptoms **eTable 4.** Vaccination Status for Cases and Controls in Analysis of Estimated Vaccine Effectiveness Against COVID-19–Related Hospitalization and Death **eTable 5.** Estimated Vaccine Effectiveness Against Laboratory-Confirmed SARS-CoV-2 Infection, Combining VA and CMS Data **eTable 6.** Estimated Vaccine Effectiveness Against Laboratory-Confirmed SARS-CoV-2 Infection by Type of Diagnostic Test
