# Supplementary material for: Coverage and Estimated Effectiveness of mRNA COVID-19 Vaccines Among US Veterans
Source: JAMA Netw Open. 2021 Oct 6;4(10):e2128391. doi: 10.1001/jamanetworkopen.2021.28391 (PMC8495523; doi:10.1001/jamanetworkopen.2021.28391)
Supplement: Supplement. — eFigure. Vaccination Exposure Classification eTable 1. Definitions of Variables eTable 2. Estimated Vaccine Effectiveness Against Laboratory-Confirmed SARS-CoV-2 Infection for Patients With Hematological Malignant Neoplasms eTable 3. Estimated Vaccine Effectiveness Against Laboratory-Confirmed SARS-CoV-2 Infection Among Veterans With COVID-19 Symptoms eTable 4. Vaccination Status for Cases and Controls in Analysis of Estimated Vaccine Effectiveness Against COVID-19–Related Hospitalization and Death eTable 5. Estimated Vaccine Effectiveness Against Laboratory-Confirmed SARS-CoV-2 Infection, Combining VA and CMS Data eTable 6. Estimated Vaccine Effectiveness Against Laboratory-Confirmed SARS-CoV-2 Infection by Type of Diagnostic Test [file jamanetwopen-e2128391-s001.pdf]

Supplemental Online Content

Young-Xu Y, Korves C, Roberts J, et al. Coverage and estimated effectiveness of mRNA COVID-19 vaccines among US veterans. *JAMA Netw Open*. 2021;4(10):e2128391. doi:10.1001/jamanetworkopen.2021.28391

eFigure. Vaccination Exposure Classification
eTable 1. Definitions of Variables
eTable 2. Estimated Vaccine Effectiveness Against Laboratory-Confirmed SARS-CoV-2 Infection for Patients With Hematological Malignant Neoplasms
eTable 3. Estimated Vaccine Effectiveness Against Laboratory-Confirmed SARS-CoV-2 Infection Among Veterans With COVID-19 Symptoms
eTable 4. Vaccination Status for Cases and Controls in Analysis of Estimated Vaccine Effectiveness Against COVID-19–Related Hospitalization and Death
eTable 5. Estimated Vaccine Effectiveness Against Laboratory-Confirmed SARS-CoV-2 Infection, Combining VA and CMS Data
eTable 6. Estimated Vaccine Effectiveness Against Laboratory-Confirmed SARS-CoV-2 Infection by Type of Diagnostic Test

This supplemental material has been provided by the authors to give readers additional information about their work.

© 2021 Young-Xu Y et al. *JAMA Network Open.*

eFigure. Vaccination Exposure Classification

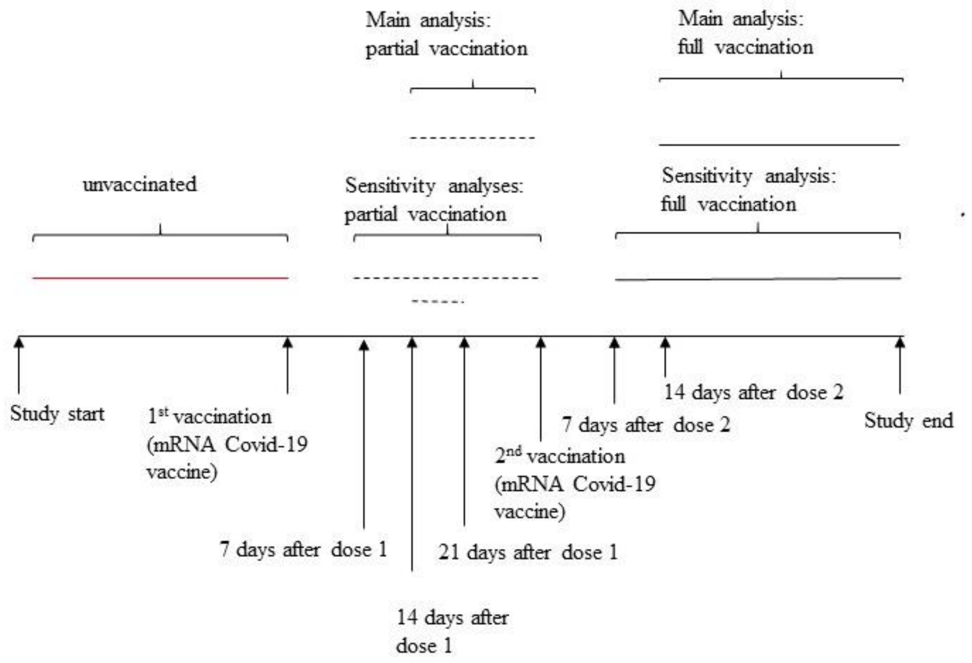

© 2021 Young-Xu Y et al. *JAMA Network Open.*

eTable 1. Definitions of Variables

Variable	Values	Definition	Timing
Age	Integer	Age as of the specimen collection date for SARS-CoV-2 lab test	Table 1: Determined at March 7, 2021; Table 2: Determined at the specimen collection date for SARS-CoV-2 lab test during the study period (December 14, 2020 to March 7, 2021)
Sex	Female/male	As defined in VHA data	Most recent available
Race/ethnicity	Non-Hispanic Black<br>Hispanic any race<br>Non-Hispanic White<br>Other	Black: non-Hispanic black<br>Hispanic: Hispanic any race<br>White: non-Hispanic white<br>Other: non-Hispanic other race, missing race, declined to state, unknown	Most recent available
Rurality	Highly Rural<br>Rural<br>Urban	VHA defined based on the Rural-Urban Community Area (RUCA) system [https://www.ruralhealth.va.gov/rural-definition.asp]	Most recent available
VHA defined priority group	1, 2, 3, 4, 5, 6, 7, 8	VA defined based on factors including military service history, disability rating and income level to identify Veterans to determine enrolment priority; 1 is the highest priority [https://www.va.gov/health-care/eligibility/priority-groups/]	Most recent available
Nursing home use	0/1	1 = Any nursing home or long-term care indicated on an inpatient admission or place of disposition	During 2 years prior to and on date of specimen collection date for SARS-CoV-2 lab test
Health and Human Services (HHS) region	Region 1<br>Region 2<br>Region 3<br>Region 4<br>Region 5<br>Region 6<br>Region 7<br>Region 8<br>Region 9<br>Region 10	Region 1: (state abbreviation) CT, ME, NH, RI, VT<br>Region 2: NJ, NY<br>Region 3: DE, DC, MD, PA, VA, WV<br>Region 4: AL, FL, GA, KY, MS, NC, SC, TN<br>Region 5: IL, IN, MI, MN, OH, WI<br>Region 6: AR, LA, NM, OK, TX<br>Region 7: IA, KS, MO, NE<br>Region 8: CO, MT, ND, SD, UT, WY<br>Region 9: AZ, CA, HI, NV<br>Region 10: AK, ID, OR, WA	Most recent available value based on location of patient's residence

© 2021 Young-Xu Y et al. *JAMA Network Open.*

Homeless	0/1	1= ICD-10 code Z59.0 or Z59.1<br>on any inpatient or outpatient record	During 2 years prior to specimen collection date for SARS-CoV-2 lab test
Low Income	0/1	1 = income less than 12,760, but not missing income<br>0 = missing or income greater than equal to 12760	During 2 years prior to specimen collection date for SARS-CoV-2 lab test
Quan-CCI	Numeric	Using ICD-10 codes from any inpatient or outpatient record, computed Charlson Comorbidity Index, Quan's version [Quan H, Sundararajan V, Halfon P, Fong A, Burnand B, Luthi JC, et al. Coding algorithms for defining comorbidities in ICD-9-CM and ICD-10 administrative data. Medical Care 2005; 43(11):1130-1139. DOI: 10.1097/01.mlr.0000182534.19832.83]	During 2 years prior to and on date of specimen collection date for SARS-CoV-2 lab test
BMI	Normal<br>Overweight/obese<br>Missing	Normal: BMI less than 26<br>Overweight/obese: BMI greater than or equal to 26	During 2 years prior to and on date of specimen collection date for SARS-CoV-2 lab test
Comorbidities		All comorbidities listed below have 1 inpatient or 2 outpatient records with the corresponding ICD-10 code	During 2 years prior to and on date of specimen collection date for SARS-CoV-2 lab test
Asthma	0/1	ICD-10 code J45*<br>ICD-10 code J44.9*<br>ICD-10 code J67.8*	
Cancer	0/1	Charlson condition definitions using ICD-10 codes	
Cancer metastatic	0/1	Charlson condition definitions using ICD-10 codes	
Coronary artery disease	0/1	ICD-10 code I 25*	
Congestive heart failure	0/1	Charlson condition definitions using ICD-10 codes	
Chronic kidney disease	0/1	ICD-10 code N18*	
Chronic obstructive pulmonary disease	0/1	Charlson condition definitions using ICD-10 codes	
Cardiovascular disease	0/1	Charlson condition definitions using ICD-10 codes	
Dementia	0/1	Charlson condition definitions using ICD-10 codes	
Diabetes mellitus with complications	0/1	Charlson condition definitions using ICD-10 codes	
Diabetes mellitus without complications	0/1	Charlson condition definitions using ICD-10 codes	
Dyslipidemia	0/1	ICD-10 code E78.5*	

© 2021 Young-Xu Y et al. *JAMA Network Open.*

HIV	0/1	Charlson condition definitions using ICD-10 codes	
Hypertension	0/1	ICD-10 code H35.03<br>ICD-10 code I10*<br>ICD-10 code I11*<br>ICD-10 code I12*<br>ICD-10 code I13*<br>ICD-10 code I15*<br>ICD-10 code I16<br>ICD-10 code I67.4	
Liver disease, mild	0/1	Charlson condition definitions using ICD-10 codes	
Liver disease, severe	0/1	Charlson condition definitions using ICD-10 codes	
Myocardial infarction (history)	0/1	Charlson condition definitions using ICD-10 codes	
Para/hemiplegia	0/1	Charlson condition definitions using ICD-10 codes	
Peptic ulcer disease	0/1	Charlson condition definitions using ICD-10 codes	
Peripheral vascular disease	0/1	Charlson condition definitions using ICD-10 codes	
Rheumatoid arthritis	0/1	Charlson condition definitions using ICD-10 codes	
Renal disease	0/1	Charlson condition definitions using ICD-10 codes	
Atrial fibrillation	0/1	ICD-10 code I48.0<br>ICD-10 code I48.11<br>ICD-10 code I48.19<br>ICD-10 code I48.21<br>ICD-10 code I48.91	
Anaphylaxis (history)	0/1	ICD-10 code Z87.892	
Arthritis	0/1	ICD-10 code M15* through M19*	
Bleeding diathesis	0/1	ICD-10 code D69.9	
Bronchiectasis	0/1	ICD-10 code J47*	
Depression	0/1	ICD-10 code F33*	
Down Syndrome	0/1	ICD-10 code Q90*	
Embolism (history)	0/1	ICD-10 code I82*<br>ICD-10 code I74*<br>ICD-10 code I26*	
Falls (history)	0/1	ICD-10 code W19*	
Gout	0/1	ICD-10 code M1A*	
Hepatitis B	0/1	ICD-10 code B19.1*	
Hepatitis C	0/1	ICD-10 code B19.2*	
Hyperlipidemia	0/1	ICD-10 code E78.2<br>ICD-10 code E78.41	

© 2021 Young-Xu Y et al. *JAMA Network Open.*

		ICD-10 code E78.49<br>ICD-10 code E78.5	
Hypersensitivity pneumonitis	0/1	ICD-10 code J67*	
Interstitial lung disease	0/1	ICD-10 code J80*<br>ICD-10 code J81*<br>ICD-10 code J82*<br>ICD-10 code J84*	
Impaired mobility	0/1	ICD-10 code Z74.01<br>ICD-10 code Z74.09	
Musculoskeletal disorder	0/1	ICD-10 code M00* through M99*	
Mycoses	0/1	ICD-10 code B35* through B49*	
Obesity	0/1	ICD-10 code E66*	
Paranoia	0/1	ICD-10 code F22*<br>ICD-10 code F60*	
Parkinson's	0/1	ICD-10 code G20*	
Pregnancy (history)	0/1	ICD-10 code Z33*	
Drug-induced anaphylaxis	0/1	ICD-10 code Z88*	
Sickle cell disease	0/1	ICD-10 code D57*	
Solid organ transplant recipient	0/1	ICD-10 code Z94*	
Stroke / transient ischemic attach	0/1	ICD-10 code I63*<br>ICD-10 code G45*	
Urinary tract infection	0/1	ICD-10 code N39.0	
Immunocompromised	0/1	ICD-10 code B20*<br>ICD-10 code B59*<br>ICD-10 code B97.3*<br>ICD-10 code D47.Z1*<br>ICD-10 code D70*<br>ICD-10 code D71*<br>ICD-10 code D72*<br>ICD-10 code D73*<br>ICD-10 code D76*<br>ICD-10 code D80*<br>ICD-10 code D81*<br>ICD-10 code D82*<br>ICD-10 code D83*<br>ICD-10 code D84*<br>ICD-10 code D89*<br>ICD-10 code M05*<br>ICD-10 code M06*<br>ICD-10 code M07*<br>ICD-10 code M08*<br>ICD-10 code M30*<br>ICD-10 code M31*<br>ICD-10 code M32*<br>ICD-10 code M33*<br>ICD-10 code M34*	

© 2021 Young-Xu Y et al. *JAMA Network Open.*

		ICD-10 code M35.0*<br>ICD-10 code M35.9*<br>ICD-10 code Q89.0*<br>ICD-10 code T45.1X1<br>ICD-10 code Z21*<br>ICD-10 code Z48.2*<br>ICD-10 code Z51.0*<br>ICD-10 code Z51.1*<br>ICD-10 code Z94*<br>[https://www.cdc.gov/vaccines/covid-19/downloads/us-flu-vaccine-effectiveness-network-protocol-508.pdf]	
Hematological malignancy	0/1	ICD-10 codes 81.70, 81.79, 81.90<br>ICD-10 codes 82.90<br>ICD-10 codes 84.10, 84.40 85.80<br>ICD-10 code C88.0<br>ICD-10 code C90.00<br>ICD-10 codes C91.00, 91.01, 91.10, 91.11, 91.40<br>ICD-10 codes C92.00, 92.01, 92.10, 92.11, 92.30<br>96.2<br>ICD-10 code D47.2	
Vaccination status	None<br>Partial<br>Full	None: until first vaccination date<br>Partial: from 7 days after the first vaccination date and before the second vaccination date<br>Full: from 7 days after the second vaccination date	Determined at the specimen collection date for SARS-CoV-2 lab test during the study period (December 14, 2020 to March 7, 2021)
Vaccine manufacturer	None<br>Moderna<br>Pfizer	None: no vaccination recorded<br>Moderna: CPT codes 91301, 0011A, 0012A or vaccination name<br>Pfizer: CPT codes 91300, 0001A, 0002A or vaccination name	Determined at date of first vaccination during the study period (December 14, 2020 to March 7, 2021)
Month of SARS-CoV-2 test	2020-Dec<br>2021-Jan<br>2021-Feb<br>2021-Mar	Month of the specimen collection date for SARS-CoV-2 lab test	Study period (December 14, 2020 to March 7, 2021)
Care setting for SARS-CoV-2 test	ED/At Admission<br>Outpatient	ED/At Admission: Specimen collection date for SARS-CoV-2 lab test on the same date as ED visit or during an inpatient stay<br>Outpatient: Specimen collection date for SARS-CoV-2 lab test on the same date as outpatient or urgent care visit	Assessed from specimen collection dates and visit dates during the study period (December 14, 2020 to March 7, 2021)
Health and Human Services (HHS) region	Region 1<br>Region 2	Region 1: (state abbreviation) CT, ME, NH, RI, VT	Most recent available value based on location of patient residency

© 2021 Young-Xu Y et al. *JAMA Network Open.*

	Region 3<br>Region 4<br>Region 5<br>Region 6<br>Region 7<br>Region 8<br>Region 9<br>Region 10	Region 2: NJ, NY<br>Region 3: DE, DC, MD, PA, VA, WV<br>Region 4: AL, FL, GA, KY, MS, NC, SC, TN<br>Region 5: IL, IN, MI, MN, OH, WI<br>Region 6: AR, LA, NM, OK, TX<br>Region 7: IA, KS, MO, NE<br>Region 8: CO, MT, ND, SD, UT, WY<br>Region 9: AZ, CA, HI, NV<br>Region 10: AK, ID, OR, WA	
Homeless	0/1	1= ICD-10 code Z59.0 or Z59.1<br>on any inpatient or outpatient record	During 2 years prior to specimen collection date for SARS-CoV-2 lab test
Low income	0/1	1 = income less than 12,760, but not missing income<br>0 = missing or income greater than equal to 12760	During 2 years prior to specimen collection date for SARS-CoV-2 lab test
Quan-CCI	Numeric	Using ICD-10 codes from any inpatient or outpatient record, computed Charlson Comorbidity Index, Quan's version [REF Quan H, Sundararajan V, Halfon P, Fong A, Burnand B, Luthi JC, et al. Coding algorithms for defining comorbidities in ICD-9-CM and ICD-10 administrative data. Medical Care 2005; 43(11):1130-1139. DOI: 10.1097/01.mlr.0000182534.19832.83]	During 2 years prior to and on date of specimen collection date for SARS-CoV-2 lab test
BMI	Normal<br>Overweight/ obese<br>Missing	Normal: BMI less than 26<br>Overweight/obese: BMI greater than or equal to 26	During 2 years prior to and on date of specimen collection date for SARS-CoV-2 lab test
CAN Score		Score to identify patients at highest risk of hospitalization and mortality. As indicated in VHA data. If missing, the score is assumed to be zero.[https://www.va.gov/HEALTHCAREEXCELLENCE/about/organization/examples/care-assessment-needs.asp]	Most recent CAN score during 2 years prior to and on date of specimen collection date for SARS-CoV-2 lab test
Mortality-1 year	Percentage of probability	Measurement of risk of mortality in next year	
Mortality-90 days	Percentage of probability	Measurement of risk of mortality in the next 90 days	
Event-1 year	Percentage of probability	Measurement of risk of any visit or event in the next year	

© 2021 Young-Xu Y et al. *JAMA Network Open.*

Event-90 days	Percentage of probability	Measurement of risk of any visit or event in the next 90 days	
Hospitalization-1 year	Percentage of probability	Measurement of risk of a hospitalization in the next year	
Hospitalization-90 days	Percentage of probability	Measurement of risk of a hospitalization in the next 90 days	
COVID-19 symptoms		Symptoms as indicated from a combination of text search on inpatient admissions, ICD-10 codes, or as indicated in the COVID Domain	+/- 14 days around and including the COVID-19 lab test specimen date.
Any	0/1	Any one of the listed symptoms	
None	0/1	Missing all of the listed symptoms	
Fever	0/1	ICD-10 code R50.9*<br>COVID domain variable fever30d	
Shortness of breath	0/1	ICD-10 code R06.02*<br>ICD-10 code R06.0*<br>ICD-10 code R09.02*<br>COVID domain variable dyspnea30d	
Cough	0/1	ICD-10 code R05*<br>COVID domain variable cough30d	
Loss of taste/smell	0/1	ICD-10 code R43.9*<br>ICD-10 code R43.8*<br>ICD-10 code R43.1*<br>ICD-10 code R43*<br>COVID domain variable lossoftaste30d	
Chills	0/1	ICD-10 code R68.83*<br>ICD-10 code R68.89*<br>COVID domain variable chills30d	
Diarrhea	0/1	ICD-10 code R19.7*<br>COVID domain variable diarrhea30d	
Sore throat	0/1	ICD-10 code J02.8*<br>ICD-10 code J02.9*<br>COVID domain variable sorethroat30d	
Myalgia	0/1	ICD-10 code M79.1*<br>COVID domain variable myalgia30d	
Vaccination (other pathogens)		Other pathogen vaccinations identified by text search or CPT code (listed below)	The year prior to the specimen collection date for SARS-CoV-2 lab test
Influenza (2019/20 season)	0/1	CPT Code between 90655 and 90659, Q2034 and Q2039<br>CPT Code 90662	

© 2021 Young-Xu Y et al. *JAMA Network Open.*

Influenza (2020/21 season)	0/1	CPT Code between 90655 and 90659, Q2034 and Q2039<br>CPT Code 90662	
Abbreviations: BMI, body mass index; CAN Score, care assessment needs score; CPT, Current Procedural Terminology; ED, emergency department; HHS, Health and Human Services; International Classification of Diseases 10th Revision; VHA, Veterans Health Administration.			

eTable 2. Estimated Vaccine Effectiveness Against Laboratory-Confirmed SARS-CoV-2 Infection for Patients with Hematological Malignant Neoplasms[a]

VE, % (95%CI)			
Full vs. no vaccination		Partial vs. no vaccination	
Unadjusted	Adjusted[b]	Unadjusted	Adjusted[b]
72 (24, 89)	69 (17, 88)	36 (-21, 66)	16 (-65, 57)

Abbreviations: BMI, body mass index; CI, confidence interval; VE, vaccine effectiveness.
[a]See *Supplemental* Table 1 for definitions of variables in this table.
[b]The adjusted variables include the following: age, BMI, cancer, congestive heart failure, chronic kidney disease, diabetes mellitus, hypertension, immunocompromised, VA priority level, race/ethnicity, sex, rurality.

© 2021 Young-Xu Y et al. *JAMA Network Open.*

eTable 3. Estimated Vaccine Effectiveness Against Laboratory-Confirmed SARS-CoV-2 Infection Among Veterans With COVID-19 Symptoms[a]

	VE, % (95%CI)			
	Full vs. no vaccination		Partial vs. no vaccination	
	Unadjusted	Adjusted[b]	Unadjusted	Adjusted[b]
Overall	94 (91, 96)	93 (89, 95)	61 (54, 66)	52 (44, 60)
Age, years				
18-64	91 (72, 97)	88 (63, 96)	62 (41, 75)	59 (37, 74)
≥65	91 (86, 94)	90 (84, 94)	45 (35, 54)	47 (37, 56)
18-79	90 (84, 94)	90 (84, 93)	55 (45, 62)	50 (40, 58)
≥80	91 (77, 96)	91 (77, 96)	51 (32, 65)	50 (30, 64)
VHA defined priority group				
1-4	94 (87, 97)	94 (88, 97)	52 (38, 63)	50 (36, 62)
5-6	85 (72, 92)	84 (70, 91)	45 (27, 58)	45 (27, 58)
7-8	89 (76, 95)	88 (72, 95)	51 (33, 64)	47 (27, 61)
Race/ethnic group				
Non-Hispanic Black	95 (83, 98)	95 (84, 98)	45 (23, 61)	40 (15, 57)
Hispanic any race			29 (-51, 67)	42 (-27, 73)
Non-Hispanic White	87 (80, 92)	86 (78, 91)	53 (43, 61)	49 (39, 58)
Other			86 (54, 95)	80 (37, 94)
Sex				
Female	81 (21, 96)	72 (-19, 94)	82 (27, 96)	82 (27, 96)
Male	91 (86, 94)	90 (85, 94)	51 (42, 58)	48 (39, 56)
Urban-rural				
Rural	90 (77, 95)	89 (76, 95)	50 (33, 63)	42 (22, 57)
Urban	91 (85, 94)	89 (83, 93)	50 (39, 58)	50 (39, 59)
BMI				
Overweight/Obese	89 (79, 94)	88 (77, 94)	46 (32, 58)	45 (30, 57)
Underlying medical conditions				
Cancer	78 (52, 90)	77 (49, 90)	41 (6, 63)	54 (27, 72)
Congestive heart failure	88 (70, 95)	85 (66, 94)	54 (32, 69)	48 (23, 65)
Chronic kidney disease	86 (70, 93)	85 (67, 93)	59 (41, 72)	56 (36, 70)
Diabetes mellitus	92 (85, 96)	91 (83, 95)	59 (47, 68)	51 (37, 62)
Hypertension	89 (82, 93)	89 (82, 93)	51 (40, 60)	52 (41, 61)
Immuno-compromised	81 (66, 89)	83 (69, 90)	39 (19, 55)	42 (22, 57)

Abbreviations: BMI, body mass index; CI, confidence interval; VE, vaccine effectiveness.
[a]See Supplemental Table 1 for definitions of variables in this table.
[b]The adjusted variables include the following: age, BMI, cancer, congestive heart failure, chronic kidney disease, diabetes mellitus, hypertension, immunocompromised, VA priority level, race/ethnicity, sex, rurality.

© 2021 Young-Xu Y et al. *JAMA Network Open.*

eTable 4. Vaccination Status for Cases and Controls in Analysis of Estimated Vaccine Effectiveness Against COVID-19–Related Hospitalization and Death

COVID-19-related hospitalization						
	Unmatched		Matched[b]			
	Cases (n=3,126)	Controls (n=322,162)	Cases		Controls	
COVID-19 vaccination status						
Full	[a]	7,867	[a]		322	
Partial	[a]	11,556	[a]		493	
None	3,041	302,739	Full v. None: 3,041	Part v. None: 3,041	Full v. None: 11,882	Part v. None: 11,971
COVID-19-related death						
	Unmatched		Matched[b]			
	Cases (n=657)	Controls (n=328,589)	Cases		Controls	
COVID-19 vaccination status						
Full	0	8,068	0		72	
Partial	13	11,814	13		96	
None	644	308,707	Full v. None: 644	Part v. None: 644	Full v. None: 2,504	Part v. None: 2,532

[a]A breakdown by full and partial vaccination status would violate federal cell size suppression policy.
[b]The number of cases and controls were different for the matched analysis for VE for full and partial vaccination.

© 2021 Young-Xu Y et al. *JAMA Network Open.*

eTable 5. Estimated Vaccine Effectiveness Against Laboratory-Confirmed SARS-CoV-2 Infection, Combining VA and CMS Data[a,b]

	VE, % (95%CI)			
	Full vs. no vaccination		Partial vs. no vaccination	
	Unadjusted	Adjusted[c]	Unadjusted	Adjusted[c]
Overall	96 (95, 97)	95 (93, 96)	71 (67, 75)	59 (52, 65)
Age, y				
18-64	91 (83, 96)	90 (80, 95)	65 (52, 75)	64 (50, 74)
≥65	93 (91, 95)	93 (90, 95)	53 (47, 59)	51 (44, 57)
18-79	92 (88, 94)	92 (88, 94)	57 (50, 63)	56 (49, 62)
≥80	93 (87, 97)	94 (88, 97)	52 (38, 62)	51 (37, 62)
VHA defined priority group				
1-4	94 (90, 96)	94 (89, 96)	59 (50, 66)	52 (41, 60)
5-6	89 (83, 93)	89 (82, 93)	46 (34, 56)	45 (32, 55)
7-8	92 (84, 96)	92 (83, 96)	45 (30, 57)	52 (39, 63)
Race/ethnic group				
Non-Hispanic Black	94 (88, 97)	94 (86, 97)	54 (40, 64)	50 (35, 62)
Hispanic any race	86 (54, 96)	85 (50, 95)	47 (0, 72)	40 (-15, 68)
Non-Hispanic White	91 (88, 94)	91 (87, 93)	53 (46, 60)	53 (46, 60)
Sex				
Female	91 (61, 98)	87 (47, 97)	87 (59, 96)	85 (51, 95)
Male	92 (90, 94)	92 (90, 94)	53 (47, 59)	53 (46, 58)
Urban-rural				
Rural	93 (87, 96)	93 (87, 96)	60 (50, 68)	59 (48, 67)
Urban	92 (89, 95)	91 (87, 94)	53 (46, 60)	49 (41, 56)
BMI				
Obese	91 (85, 95)	90 (83, 94)	44 (32, 53)	45 (35, 55)
Underlying medical conditions				
Cancer	82 (66, 91)	84 (70, 92)	53 (32, 68)	56 (36, 70)
Congestive heart failure	87 (76, 92)	89 (80, 94)	64 (50, 74)	66 (52, 75)
Chronic kidney disease	89 (82, 94)	90 (82, 94)	56 (42, 67)	53 (38, 65)
Diabetes mellitus	92 (88, 95)	92 (88, 95)	59 (51, 66)	57 (48, 64)
Hypertension	92%(88, 94)	91 (87, 94)	56 (49, 62)	54 (47, 61)
Immunocompromised	85 (77, 91)	85 (77, 91)	44 (29, 56)	50 (36, 61)

Abbreviations: BMI, body mass index; CI, confidence interval; CMS, Centers for Medicare and Medicaid Services; ICD-10, International Classification of Diseases 10th Revision; VA, Veterans Affairs; VE, vaccine effectiveness.

[a]Since 2020, for VHA enrolled patients with Medicare who have either tested positive for SARS-Cov-2 at a VHA facility or who have a COVID-19 medical claim in Medicare (ICD-10 codes B97.29 [“Other coronavirus”] and U07.1 [“COVID-19”] diagnosis code in inpatient, skilled nursing facility, institutional outpatient, hospice, and carrier/Part B files), the VHA receives the patients’ Medicare data with a 4-5 week lag time [REF https://vaww.virec.research.va.gov/VACMS/Medicare/COVID19-Data.htm].

[b]See Supplemental Table 1 for definitions of variables in this table.

[c]The adjusted variables include the following: age, BMI, cancer, congestive heart failure, chronic kidney disease, diabetes mellitus, hypertension, immunocompromised, VA priority level, race/ethnicity, sex, rurality.

© 2021 Young-Xu Y et al. *JAMA Network Open.*

eTable 6. Estimated Vaccine Effectiveness Against Laboratory-Confirmed SARS-CoV-2 Infection by Type of Diagnostic Test[a]

	VE, % (95%CI)			
	Full vs. no vaccination		Partial vs. no vaccination	
	Unadjusted	Adjusted[b]	Unadjusted	Adjusted[b]
PCR test	96 (94, 97)	93 (90, 95)	71 (67, 74)	59 (53, 64)
Antigen test	96 (88, 99)	97 (91, 99)	67 (46, 80)	70 (48, 82)

Abbreviations: BMI, body mass index; CI, confidence interval; VE, vaccine effectiveness; VHA, Veterans Health Affairs.
[a]See Supplemental Table 1 for definitions of variables in this table.
[b]The adjusted variables include the following: age, BMI, cancer, congestive heart failure, chronic kidney disease, diabetes mellitus, hypertension, immunocompromised, VA priority level, race/ethnicity, sex, rurality.

© 2021 Young-Xu Y et al. *JAMA Network Open.*
